# Genetic polymorphisms of estrogen receptor genes are associated with breast cancer susceptibility in Chinese women

**DOI:** 10.1186/s12935-019-0727-z

**Published:** 2019-01-08

**Authors:** Zhijun Dai, Tian Tian, Meng Wang, Tielin Yang, Hongtao Li, Shuai Lin, Qian Hao, Peng Xu, Yujiao Deng, Linghui Zhou, Na Li, Yan Diao

**Affiliations:** 1 Department of Breast Surgery, Guangzhou Women and Children’s Medical Center, Guangzhou Medical University, Guangzhou, 510623 Guangdong China; 2 Department of Oncology, The Second Affiliated Hospital of Xi’an Jiaotong University, Xi’an, 710004 China; 3 School of Life Science and Technology, Xi’an Jiaotong University, Xi’an, 710049 China; 4 Department of Breast Head and Neck Surgery, The 3rd Affiliated Teaching Hospital of Xinjiang Medical University (Affiliated Tumor Hospital), Urumqi, 830000 China

**Keywords:** Estrogen receptor genes, Polymorphism, Breast cancer, Susceptibility

## Abstract

**Background:**

Estrogen exposure is a widely known risk factor for BC. And the interaction of estrogen with estrogen receptor (ER) plays an important role in breast cancer development. This case–control study aims to assess the association of genetic polymorphisms in the estrogen receptor genes with breast cancer (BC) susceptibility in Chinese Han women.

**Methods:**

Four polymorphisms (rs2881766, rs9383951, rs9340799 in *ESR1* and rs3020449 in *ESR2*) were genotyped in 459 patients and 549 healthy controls using the Sequenom MassARRAY method. Odds ratio (OR) and 95% confidence intervals (95% CI) were calculated to evaluate the associations. False-positive report probability (FPRP) was utilized to examine the noteworthiness of significant findings.

**Results:**

We observed that rs2881766 was associated with a decreased BC risk (GG vs. TT: OR = 0.63, 95% CI = 0.44–0.91; GG vs. TT/GT: OR = 0.68, 95% CI = 0.49–0.95), while rs3020449 was associated with an increased risk of BC (CT vs. TT: OR = 1.58, 95% CI = 1.21–2.06; CT/CC vs. TT: OR = 1.54, 95% CI = 1.20–1.98; TT/CC vs. CT: OR = 1.48, 95% CI = 1.15–1.90). The other two polymorphisms have no relation with BC susceptibility. In addition, rs2881766 was correlated with lymph node metastasis and ER expression, and rs3020449 was related to tumor size, histological grade and ER expression. The values of false-positive report probability indicated that the significant associations of BC risk with both rs2881766 and rs3020449 were noteworthy.

**Conclusions:**

Our study suggests that polymorphisms rs2881766 and rs3020449 in estrogen receptor genes were associated with BC susceptibility as well as clinical features in Chinese women. These findings need further validation in a large population.

**Electronic supplementary material:**

The online version of this article (10.1186/s12935-019-0727-z) contains supplementary material, which is available to authorized users.

## Background

Breast cancer (BC), which had approximately 1.7 million new cases and 0.5 million deaths in 2012, has become the most common cancer type and the leading cause of global cancer death among females [1]. In Chinese women, BC also holds the highest incidence, accounting for 15% of all new cancers and 7% malignancy deaths [2]. BC is a complex heterogeneous disease. Both genetic and environmental factors are involved in the occurrence of BC [3].

Estrogen exposure is a widely known risk factor for BC. And the interaction of estrogen with estrogen receptor (ER), which can alter the expression of downstream genes, plays an important role in breast cancer development [4]. ER alpha (ERα) and beta (ERβ) are the main forms of ER. They were encoded by the gene estrogen receptor 1 (*ESR1*) and estrogen receptor 2 (*ESR2*) respectively. And evidence showed that genetic variants in these two genes were associated with BC susceptibility [5–8]. Polymorphisms in *ESR1* have been investigated in a number of studies. The pooled results demonstrated that the AA genotype of rs2228480 was correlated with a lower risk of BC in Caucasians. The C allele of the rs3798577 decreased BC risk in Asians while increased BC risk in Caucasians [5]. The TT/TC genotype of rs2234693 was related to high risk of BC [8]. In addition, meta-analysis of the relationship between *ESR2* polymorphisms and BC susceptibility showed that the polymorphism rs4986938 was associated with reduced BC risk in overall population under both dominant and heterozygous models [6].

In the present study, four single nucleotide polymorphisms (SNPs) in the two estrogen receptor genes were selected to be studied. The effects of these SNPs (rs2881766, rs9383951, rs9340799 in *ESR1* and rs3020449 in *ESR2*) in BC risk in Chinese population were either seldom being explored or the conclusions were still controversial. Therefore, we conducted a hospital-based case–control study to explore the association of the four polymorphisms with BC susceptibility in Chinese Han women.

## Methods

### Study population

BC patients treated at the Department of Oncology, the Second Affiliated Hospital of Xi’an Jiaotong University (Xi’an, China) were enrolled as cases [9]. Women who came to the hospital for a checkup during the same period of time were recruited to form the control group. The cases were newly diagnosed and confirmed by histology or pathology. None of them had received chemotherapy, endocrinotherapy or radiotherapy before surgery. Patients with other types of cancer were excluded. The controls were healthy individuals without any history of a tumor or chronic diseases. All of the participants were Han population from Northwest China and have no relation to each other. Clinical information was collected from the medical records of the study subjects.

### Sample collection and genotyping

Peripheral blood samples were collected in tubes coated with EDTA and were stored at − 80 °C. Genomic DNA was extracted from whole blood using the Universal Genomic DNA Extraction Kit (TaKaRa Bio Inc., Japan). DNA concentration was measured with the UV/VIS spectrophotometer (DU530, Beckman Instruments, USA). Four tag-SNPs (rs2881766, rs9383951, rs9340799 and rs3020449) were selected from The Single Nucleotide Polymorphism database (dbSNP). A multiplexed SNP MassEXTEND assay was designed by Sequenom MassARRAY Assay Design V3.0 Software (Agena Bioscience Inc., USA). And SNP genotypes was detected using Sequenom MassARRAY RS1000 [10, 11]. The primers of the selected SNPs were listed in Additional file 1: Table S1. Data was analyzed with Sequenom Typer Software (version 4.0, USA).

### Statistical analysis

The Student *t* test and χ^2^-test were adopted to compare the differences in basic characteristics between cases and controls. Hardy–Weinberg equilibrium (HWE) for selected SNPs in controls and the differences in genotypes distribution between cases and controls were examined by χ^2^-test. We evaluated the associations of the four SNPs with BC risk in codominant, dominant, recessive, and overdominant models. Odds ratios (ORs) with 95% confidence intervals (CIs) were calculated by multivariate logistic regression, with adjustment for age and body mass index (BMI). All the tests were two-tailed and *P*-value < 0.05 was considered statistically significant. Akaike’s information criterion (AIC) and Bayesian information criterion (BIC) values were used to determine the best-fit model for each SNP. We then conducted False-positive report probability (FPRP) analysis to examine whether the significant results were noteworthy. The prior probability of 0.1 was set to detect the noteworthiness for OR of 1.50/0.67 and 0.25 was determined as a FPRP cut-off value as described in previous studies [12, 13]. All data were analyzed using SPSS software (version 22.0, IBM Corporation, USA).

## Results

### Basic information of the study population and SNPs

A total of 1008 subjects including 459 BC patients and 549 healthy individuals were enrolled in this study. As shown in Table 1, no significant difference was observed between the case and control groups in the distribution of age, menopausal status, procreative times and BMI. It suggested that the cases and controls in this study were matched adequately on basic characteristics. The basic information of the four SNPs was presented in Table 2. The genotypic frequencies of all the selected SNPs in controls were in accordance with HWE. The frequency distribution of clinicopathological features in BC patients was shown in Additional file 1: Table S2.

**Table 1:** The basic characteristics of cases and controls

Characteristics	Cases (459)	Controls (549)	*P*-value
Age (mean ± SD)	49.09 ± 11.02	48.80 ± 8.28	0.610
*Menopausal status*			0.376
Premenopausal	237	267	
Postmenopausal	222	282	
*Procreative times*			0.657
< 2	242	298	
≥ 2	217	251	
BMI (kg/m^2^, mean ± SD)	23.06 ± 2.92	22.45 ± 2.53	0.274

*BMI* body mass index

**Table 2:** The basic information of selected SNPs

Rs number	Gene symbol	Allele	Chromosome position	MAF	*P*_HWE_
rs2881766	*ESR1*	G/T	Chr6:151797984	0.452	0.556
rs9383951	*ESR1*	C/G	Chr6:151974478	0.068	0.662
rs9340799	*ESR1*	A/G	Chr6:151842246	0.281	0.797
rs3020449	*ESR2*	C/T	Chr14:64306674	0.484	0.501

### Association between *ESR1* and *ESR2* SNPs with BC risk

Among the three *ESR1* polymorphisms, rs2881766 was found to reduce BC risk under homozygous model (GG vs. TT: OR = 0.63, 95% CI = 0.44–0.91) and recessive model (GG vs. TT/GT: OR = 0.68, 95% CI = 0.49–0.95). The model with the lowest AIC and BIC values was considered as the best-fit model. For rs2881766, the recessive model might serve as the best-fit model. The other two SNPs (rs9383951 and rs9340799) were not related to BC susceptibility. As for the *ESR2* polymorphism rs3020449, an increased risk of BC was found in heterozygous model (CT vs. TT: OR = 1.58, 95% CI = 1.21–2.06), dominant model (CT/CC vs. TT: OR = 1.54, 95% CI = 1.20–1.98), and overdominant model (TT/CC vs. CT: OR = 1.48, 95% CI = 1.15–1.90). And the AIC and BIC values suggested the dominant model may be the best-fit model for rs3020449 (Table 3).

**Table 3:** The associations of *ESR1* and *ESR2* polymorphisms with breast cancer risk (adjusted by age and BMI)

Model	Genotype	Cases (n, %)	Control (n, %)	OR (95% CI)	*P*-value	AIC	BIC
*rs2881766*		459	549				
Codominant	TT	173 (37.7%)	178 (32.4%)	1.00	–	–	–
	GT	218 (47.5%)	260 (47.4%)	0.87 (0.66–1.15)	–	–	–
	GG	68 (14.8%)	111 (20.2%)	*0.63 (0.44*–*0.91)*	*0.046*	1387.9	1412.5
Dominant	TT	173 (37.7%)	178 (32.4%)	1.00	–	–	–
	GT/GG	286 (62.3%)	371 (67.6%)	0.80 (0.62–1.04)	0.091	1389.2	1408.9
Recessive	TT/GT	391 (85.2%)	438 (79.8%)	1.00	–	–	–
	GG	68 (14.8%)	111 (20.2%)	*0.68 (0.49*–*0.95)*	0.022*	1386.9	1406.5
Overdominant	TT/GG	241 (52.5%)	289 (52.6%)	1.00	–	–	–
	GT	218 (47.5%)	260 (47.4%)	1.02 (0.79–1.30)	0.9	1392.1	1411.7
*rs9383951*		458	549				
Codominant	GG	379 (82.8%)	442 (80.5%)	1.00	–	–	–
	GC	76 (16.6%)	102 (18.6%)	0.89 (0.64–1.23)	–	–	–
	CC	3 (0.7%)	5 (0.9%)	0.77 (0.18–3.28)	0.74	1391.7	1416.3
Dominant	GG	379 (82.8%)	442 (80.5%)	1.00	–	–	–
	GC/CC	79 (17.2%)	107 (19.5%)	0.88 (0.64–1.22)	0.45	1389.8	1409.4
Recessive	GG/GC	455 (99.3%)	544 (99.1%)	1.00	–	–	–
	CC	3 (0.7%)	5 (0.9%)	0.79 (0.19–3.35)	0.75	1390.3	1409.9
Overdominant	GG/CC	382 (83.4%)	447 (81.4%)	1.00	–	–	–
	GC	76 (16.6%)	102 (18.6%)	0.89 (0.64–1.24)	0.48	1389.9	1409.5
*rs9340799*		459	549				
Codominant	AA	289 (63%)	349 (63.6%)	1.00	–	–	–
	GA	144 (31.4%)	179 (32.6%)	0.97 (0.74–1.27)	–	–	–
	GG	26 (5.7%)	21 (3.8%)	1.44 (0.79–2.62)	0.44	1392.5	1417.0
Dominant	AA	289 (63%)	349 (63.6%)	1.00	–	–	–
	GA/GG	170 (37%)	200 (36.4%)	1.02 (0.79–1.32)	0.89	1392.1	1411.7
Recessive	AA/GA	433 (94.3%)	528 (96.2%)	1.00	–	–	–
	GG	26 (5.7%)	21 (3.8%)	1.46 (0.81–2.63)	0.21	1390.5	1410.2
Overdominant	AA/GG	315 (68.6%)	370 (67.4%)	1.00	–	–	–
	GA	144 (31.4%)	179 (32.6%)	0.94 (0.72–1.23)	0.67	1391.9	1411.6
*rs3020449*		459	549				
Codominant	TT	176 (38.3%)	267 (48.6%)	1.00	–	–	–
	CT	230 (50.1%)	224 (40.8%)	*1.58 (1.21*–*2.06)*	–	–	–
	CC	53 (11.6%)	58 (10.6%)	1.39 (0.91–2.11)	*0.003*	1382.5	1407.1
Dominant	TT	176 (38.3%)	267 (48.6%)	1.00	–	–	–
	CT/CC	283 (61.7%)	282 (51.4%)	*1.54 (1.20*–*1.98)*	8e−04*	1380.8	1400.5
Recessive	TT/CT	406 (88.5%)	491 (89.4%)	1.00	–	–	–
	CC	53 (11.6%)	58 (10.6%)	1.10 (0.74–1.64)	0.64	1391.9	1411.5
Overdominant	TT/CC	229 (49.9%)	325 (59.2%)	1.00	–	–	–
	CT	230 (50.1%)	224 (40.8%)	*1.48 (1.15*–*1.90)*	*0.002*	1382.8	1402.5

The significant ORs and 95%CIs were presented in italic

*OR* odds ratio, *95% CI* confidence interval, *AIC* Akaike’s information criterion, *BIC* Bayesian information criterion

* *P*-value of best-fit model

### FPRP analysis results

FPRP was adopted to assess the noteworthiness of the significant associations between the selected SNPs and BC risk. At the prior probability of 0.1 and FPRP cut-off value of 0.25, the associations between ESR2 rs3020449 and BC risk remained noteworthy in the three models (FPRP = 0.018, 0.016, and 0.034 respectively). In addition, the significant decrease of BC risk in carrier of ESR1 rs2881766 GG genotype was noteworthy (FPRP = 0.250 under GG vs.TT) (Table 4).

**Table 4:** FPRP analysis for the significant associations of *ESR1* and *ESR2* SNPs with BC risk

Model	OR (95% CI)	Prior probability			
		0.25	0.1	0.01	0.001
*ESR1 rs2881766*					
GG vs.TT	0.63 (0.44–0.91)	0.100*	0.250*	0.786	0.974
GG vs.TT/GT	0.68 (0.49–0.95)	0.118*	0.286	0.815	0.978
*ESR2 rs3020449*					
CT vs.TT	1.58 (1.21–2.06)	0.006*	0.018*	0.170*	0.674
CT/CC vs.TT	1.54 (1.20–1.98)	0.005*	0.016*	0.152*	0.644
CT vs.TT/CC	1.48 (1.15–1.90)	0.011*	0.034*	0.277	0.795

The significant ORs and 95%CIs were presented in italic

* Noteworthiness at the 0.25 level of FPRP

### Relationship of rs2881766 and rs3020449 with clinicopathological features of BC

We further explored the relationship of *ESR1* rs2881766 and *ESR2* rs3020449 with BC clinicopathological features. The results showed that the GG genotype of rs2881766 was negatively correlated with lymph node metastasis (GG vs.TT: OR = 0.47, 95% CI = 0.27–0.83; GG vs.TT/GT: OR = 0.54, 95% CI = 0.32–0.91) and ER+ status (GG vs.TT: OR = 0.52, 95% CI = 0.29–0.91; GG vs.TT/GT: OR = 0.53, 95% CI = 0.32–0.90) (Table 5).

**Table 5:** The associations between *ESR1* rs2881766 and clinicopathological features of breast cancer

Variables	Genotype			OR (95% CI)			
	TT	GT	GG	GG vs.TT	GT vs.TT	GT/GG vs.TT	GG vs. TT/GT
*Tumor size (cm)*							
< 2	55	72	25				
≥ 2	118	146	43	0.80 (0.45–1.44)	0.95 (0.62–1.45)	0.91 (0.61–1.36)	0.83 (0.48–1.41)
*LN metastasis*							
No	60	88	36				
Yes	113	130	32	*0.47 (0.27–0.83)*	0.78 (0.52–1.19)	0.69 (0.47–1.03)	*0.54 (0.32–0.91)*
*Histological grade*							
SBR 1–2	89	117	38				
SBR 3	84	101	30	0.84 (0.48–1.47)	0.91 (0.61–1.36)	0.90 (0.61–1.31)	0.88 (0.52–1.48)
*Venous invasion*							
None–little	106	140	46				
Moderate-severe	67	78	22	0.76 (0.42–1.37)	0.88 (0.58–1.33)	0.85 (0.58–1.26)	0.81 (0.47–1.40)
*ER*							
(−)	71	92	39				
(+)	102	126	29	*0.52 (0.29–0.91)*	0.95 (0.64–1.43)	0.82 (0.56–1.21)	*0.53 (0.32–0.90)*
*PR*							
(−)	75	100	33				
(+)	98	118	35	0.81 (0.46–1.42)	0.90 (0.60–1.35)	0.88 (0.60–1.29)	0.86 (0.51–1.44)
*Her-2*							
(−)	128	155	47				
(+)	45	63	21	1.27 (0.69–2.35)	1.16 (0.74–1.81)	1.18 (0.77–1.81)	1.17 (0.67–2.05)
*Ki–67 (%)*							
< 14	109	140	45				
≥ 14	64	78	23	0.87 (0.48–1.57)	0.95 (0.63–1.44)	0.93 (0.63–1.38)	0.90 (0.52–1.54)

The significant ORs and 95%CIs were presented in italic

*LN* lymph node, *ER* estrogen receptor, *PR* progesterone receptor, *Her-2* human epidermal growth factor receptor 2, *SBR* Scarff, Bloom and Richardson tumor grade, *OR* odds ratio, *95% CI* confidence interval

The rs3020449 was related to a larger tumor size under heterozygous and dominant model (CT vs. TT: OR = 1.60, 95% CI = 1.06–2.43; CT/CC vs. TT: OR = 1.55, 95% CI = 1.05–2.31). In addition, the CC genotype **of** rs3020449 was associated with higher histological grade (CC vs. TT: OR = 1.90, 95% CI = 1.02–3.54) as well as ER positive status (CC vs. TT: OR = 2.16, 95% CI = 1.12–4.17; CC vs. TT/CT: OR = 1.96, 95% CI = 1.05–3.63) (Table 6).

**Table 6:** The associations between *ESR2* rs3020449 and clinicopathological features of breast cancer

Variables	Genotype			OR (95% CI)			
	TT	CT	CC	CC vs.TT	CT vs.TT	CT/CC vs.TT	CC vs. TT/CT
*Tumor size (cm)*							
< 2	69	66	17				
≥ 2	107	164	36	1.37 (0.71–2.62)	*1.60 (1.06–2.43)*	*1.55 (1.05–2.31)*	1.057 (0.57–1.95)
*LN metastasis*							
No	74	90	20				
Yes	102	140	33	1.20 (0.64–2.25)	1.13 (0.76–1.68)	1.14 (0.78–1.67)	1.12 (0.62–2.02)
*Histological grade*							
SBR 1–2	101	121	22				
SBR 3	75	109	31	*1.90 (1.02–3.54)*	1.21 (0.82–1.80)	1.32 (0.90–1.93)	1.70 (0.95–3.04)
*Venous invasion*							
None-little	114	146	32				
Moderate-severe	62	84	21	1.21 (0.64–2.27)	1.06 (0.70–1.59)	1.08 (0.73–1.61)	1.17 (0.65–2.10)
*ER*							
(−)	85	101	16				
(+)	91	129	37	*2.16 (1.12–4.17)*	1.19 (0.80–1.77)	1.33 (0.91–1.94)	*1.96 (1.05–3.63)*
*PR*							
(−)	83	102	23				
(+)	93	128	30	1.16 (0.63–2.16)	1.12 (0.76–1.66)	1.13 (0.77–1.65)	1.09 (0.61–1.94)
*Her-2*							
(−)	120	175	35				
(+)	56	55	18	1.10 (0.57–2.11)	0.67 (0.43–1.04)	0.74 (0.49–1.13)	1.37 (0.74–2.51)
*Ki–67 (%)*							
< 14	112	153	29				
≥ 14	64	77	24	1.45 (0.78–2.70)	0.88 (0.58–1.33)	0.97 (0.66–1.44)	1.56 (0.87–2.77)

The significant ORs and 95%CIs were presented in italic

*LN* axillary lymph node, *ER* estrogen receptor, *PR* progesterone receptor, *Her-2* human epidermal growth factor receptor 2, *SBR* Scarff, Bloom and Richardson tumor grade, *OR* odds ratio; *95% CI* confidence interval

## Discussion

Genetic variation is an important risk factor for BC [14]. Single nucleotide polymorphism is the most frequent variation in the genome. Genome-wide association study (GWAS) has identified numerous BC susceptibility loci in tumor-related genes such as *FGFR2, TOX3, TP53, PTEN, MAP3K1, c-MYC, LSP1,* and *CASP8* [14, 15]. Estrogen plays a critical role in the development of breast cancer [4]. Its effect on the breast epithelium is mediated by ER. The two major forms of ER (ERα and ERβ) are encoded by two separate genes, *ESR1* located on Chr6 and *ESR2* located on Chr14. And genetic polymorphisms in these two genes were reported to associate with BC susceptibility, including the extensively studied SNPs such as rs2228480, rs3798577, rs2077647, rs2234693 in *ESR1* and rs4986938, rs1256049 in *ESR2* as well as less common SNPs such as rs3020314, rs1514348, rs1062577, rs1271572, rs1256054 and rs2987983 [5–8, 16–19].

In this study, we investigated the associations of BC risk with four SNPs in the estrogen receptor genes *ESR1* and *ESR2*. SNP rs2881766 is located in the promoter region of *ESR1*. This polymorphism was reported to increase BC risk in Korean women in a previously [20]. However, another study conducted in Chinese population showed that the GG genotype of this SNP decreases BC risk in menarche > 13-year-old while increases BC risk in menarche ≤ 13-year-old subgroup [21]. Our results indicated that the carriers of rs2881766 GG genotype had a lower risk of BC compared with the TT and GT genotype carriers. The discrepancy between our results and previous studies may caused by different sample sizes and the effects of confounding factors such as age, ethnicity and environmental effects. Moreover, the GG genotype may be a protective factor of lymph node metastasis for BC patients. And patients with rs2881766 GG genotype are more likely to have less expression of ER. SNP rs9340799 and rs9383951 are located in the first and fifth intron of *ESR1* respectively [8, 22]. The association between rs9340799 (also known as Xbal) and BC risk has been evaluated in several studies before, but the conclusions were debatable. A few studies suggested this polymorphism can affect BC risk [23–25], whereas others did not find any relationship between this polymorphism and BC susceptibility [8, 18, 26, 27]. There is only one previous study of rs9383951and BC risk, but no significant association was found. Our study indicated that neither rs9340799 nor rs9383951 were related to BC risk, which is in line with most of previous studies. SNP rs3020449 is located in the promoter region of the *ESR2* gene [7]. Two previous studies investigated the effect of this SNP on BC risk, but neither one observed significant association [19, 28]. In contrast, our results suggested that the CT genotype of rs3020449 increased the risk of BC. In addition, BC patients with CT genotype tend to have a larger tumor size compared with TT genotype carriers. Furthermore, patients with CC genotype may have a greater tumor grade and higher expression of ER.

FPRP analysis is an effective approach to verify the noteworthiness of significant findings. In this study, we adopted a relatively stringent cut-off value for FPRP. The FPRP value of the significant association between *ESR2* rs3020449 and BC risk was much lower than the threshold, suggesting that our findings of this SNP were noteworthy and authentic. The significant association of *ESR1* rs2881766 with BC risk was noteworthy only in homozygous model. But actually, FPRP value of significant association in another model was still quite small (< 0.3). Hence, we believe our findings for this polymorphism were credible to some extent.

## Conclusions

In conclusion, the current study suggests that the *ESR1* rs2881766 decreases BC risk while *ESR2* rs3020449 increases BC risk in Chinese women. Future large studies are required to validate these findings. The possible mechanisms underlying the associations also need to be explored.

## Additional file

**Additional file 1. Table S1:** Primers used for the study. **Table S2**: Frequency distribution of clinicopathological features in breast cancer patients.

## Abbreviations

ER: estrogen receptor
BC: breast cancer
SNP: single nucleotide polymorphism
OR: odds ratio
CI: confidence interval
HWE: Hardy–Weinberg equilibrium
AIC: Akaike’s information criterion
BIC: Bayesian information criterion
FPRP: false-positive report probability

## References

[1] Torre LA, Bray F, Siegel RL, Ferlay J, Lortet-Tieulent J, Jemal A (2015). Global cancer statistics, 2012. Cancer J Clin.

[2] Chen W, Zheng R, Baade PD, Zhang S, Zeng H, Bray F, Jemal A, Yu XQ, He J (2016). Cancer statistics in China. Cancer J Clin.

[3] Fletcher O, Dudbridge F (2014). Candidate gene-environment interactions in breast cancer. BMC Med.

[4] Yager JD, Davidson NE (2006). Estrogen carcinogenesis in breast cancer. N Engl J Med.

[5] Li T, Zhao J, Yang J, Ma X, Dai Q, Huang H, Wang L, Liu P (2016). A meta-analysis of the association between ESR1 genetic variants and the risk of breast cancer. PLoS ONE.

[6] Yu KD, Rao NY, Chen AX, Fan L, Yang C, Shao ZM (2011). A systematic review of the relationship between polymorphic sites in the estrogen receptor-beta (ESR2) gene and breast cancer risk. Breast Cancer Res Treat.

[7] Jahandoost S, Farhanghian P, Abbasi S (2017). The effects of sex protein receptors and sex steroid hormone gene polymorphisms on breast cancer Risk. J Natl Med Assoc.

[8] Zhang Y, Zhang M, Yuan X, Zhang Z, Zhang P, Chao H, Jiang L, Jiang J (2015). Association between ESR1 PvuII, XbaI, and P325P polymorphisms and breast cancer susceptibility: a meta-analysis. Med Sci Monit.

[9] Liu D, Wang M, Tian T, Wang XJ, Kang HF, Jin TB, Zhang SQ, Guan HT, Yang PT, Liu K (2017). Genetic polymorphisms (rs10636 and rs28366003) in metallothionein 2A increase breast cancer risk in Chinese Han population. Aging.

[10] Gabriel S, Ziaugra L, Tabbaa D: SNP genotyping using the Sequenom MassARRAY iPLEX platform. Curr Prot Hum Genet 2009, Chapter 2:Unit 2.12. 10.1002/0471142905.hg0212s60 19170031

[11] Wang M, Tian T, Ma X, Zhu W, Guo Y, Duan Z, Fan J, Lin S, Liu K, Zheng Y (2017). Genetic polymorphisms in caveolin-1 associate with breast cancer risk in Chinese Han population. Oncotarget.

[12] Wacholder S, Chanock S, Garcia-Closas M, El Ghormli L, Rothman N (2004). Assessing the probability that a positive report is false: an approach for molecular epidemiology studies. J Natl Cancer Inst.

[13] Lai MC, Yang Z, Zhou L, Zhu QQ, Xie HY, Zhang F, Wu LM, Chen LM, Zheng SS (2012). Long non-coding RNA MALAT-1 overexpression predicts tumor recurrence of hepatocellular carcinoma after liver transplantation. Med Oncol (Northwood, London, England).

[14] Mavaddat N, Antoniou AC, Easton DF, Garcia-Closas M (2010). Genetic susceptibility to breast cancer. Mol Oncol.

[15] Mahdi KM, Nassiri MR, Nasiri K (2013). Hereditary genes and SNPs associated with breast cancer. Asian Pac J Cancer Prev.

[16] Lipphardt MF, Deryal M, Ong MF, Schmidt W, Mahlknecht U (2013). ESR1 single nucleotide polymorphisms predict breast cancer susceptibility in the central European Caucasian population. Int J Clin Exp Med.

[17] Dehghan Z, Sadeghi S, Tabatabaeian H, Ghaedi K, Azadeh M, Fazilati M, Bagheri F (2017). ESR1 single nucleotide polymorphism rs1062577 (c.*3804T > A) alters the susceptibility of breast cancer risk in Iranian population. Gene.

[18] Ghali R, Al-Mutawa MA, Al-Ansari AK, Zaied S, Bhiri H, Mahjoub T, Almawi WY (2018). Differential association of ESR1 and ESR2 gene variants with the risk of breast cancer and associated features: a case–control study. Gene.

[19] Chen L, Liang Y, Qiu J, Zhang L, Chen X, Luo X, Jiang J (2013). Significance of rs1271572 in the estrogen receptor beta gene promoter and its correlation with breast cancer in a southwestern Chinese population. J Biomed Sci.

[20] Son BH, Kim MK, Yun YM, Kim HJ, Yu JH, Ko BS, Kim H, Ahn SH (2015). Genetic polymorphism of ESR1 rs2881766 increases breast cancer risk in Korean women. J Cancer Res Clin Oncol.

[21] Chen L, Kang H, Jin GJ, Chen X, Zhang QY, Lao WT, Li R (2016). The association between a novel polymorphism (rs1062577) in ESR1 and breast cancer susceptibility in the Han Chinese women. Gynecol Endocrinol.

[22] Long J, Cai Q, Sung H, Shi J, Zhang B, Choi JY, Wen W, Delahanty RJ, Lu W, Gao YT (2012). Genome-wide association study in east Asians identifies novel susceptibility loci for breast cancer. PLoS Genet.

[23] Shin A, Kang D, Nishio H, Lee MJ, Park SK, Kim SU, Noh DY, Choe KJ, Ahn SH, Hirvonen A (2003). Estrogen receptor alpha gene polymorphisms and breast cancer risk. Breast Cancer Res Treat.

[24] Wang J, Higuchi R, Modugno F, Li J, Umblas N, Lee J, Lui LY, Ziv E, Tice JA, Cummings SR (2007). Estrogen receptor alpha haplotypes and breast cancer risk in older Caucasian women. Breast Cancer Res Treat.

[25] Madeira KP, Daltoe RD, Sirtoli GM, Carvalho AA, Rangel LB, Silva IV (2014). Estrogen receptor alpha (ERS1) SNPs c454-397T > C (PvuII) and c454-351A > G (XbaI) are risk biomarkers for breast cancer development. Mol Biol Rep.

[26] Sun MY, Du HY, Zhu AN, Liang HY, de Garibay GR, Li FX, Li M, Yang XX (2015). Genetic polymorphisms in estrogen-related genes and the risk of breast cancer among Han Chinese women. Int J Mol Sci.

[27] Li N, Dong J, Hu Z, Shen H, Dai M (2010). Potentially functional polymorphisms in ESR1 and breast cancer risk: a meta-analysis. Breast Cancer Res Treat.

[28] Treeck O, Elemenler E, Kriener C, Horn F, Springwald A, Hartmann A, Ortmann O (2009). Polymorphisms in the promoter region of ESR2 gene and breast cancer susceptibility. J Steroid Biochem Mol Biol.

