# Supplementary material for: Genetic polymorphisms of estrogen receptor genes are associated with breast cancer susceptibility in Chinese women
Source: Cancer Cell Int. 2019 Jan 8;19:11. doi: 10.1186/s12935-019-0727-z (PMC6325673; doi:10.1186/s12935-019-0727-z)
Supplement: Supplementary file 1 — Additional file 1. Table S1: Primers used for the study. Table S2: Frequency distribution of clinicopathological features in breast cancer patients. [file 12935_2019_727_MOESM1_ESM.docx]

**Table S1** Primers used for the study.

| **SNP_ID** | **1st-PCRP** | **2nd-PCRP** | **UEP_SEQ** |
| --- | --- | --- | --- |
| rs2881766 | ACGTTGGATGAAATGCCAGTTTGCTCAAGG | ACGTTGGATGCTACATATGAGGTGGGAAAG | CATTCCTATAAACTGCAGACT |
| rs9383951 | ACGTTGGATGACACTGAAACACGTGGTCTG | ACGTTGGATGGTAAGTCCTAGGCTCTTTCC | TCCTCTCAGGTCAATATCA |
| rs9340799 | ACGTTGGATGCTGAGTTCCAAATGTCCCAG | ACGTTGGATGTTAGAGACCAATGCTCATCC | CCAATGCTCATCCCAACTC |
| rs3020449 | ACGTTGGATGACTCCGGTTGGTCTTAATGC | ACGTTGGATGGATCAGTAGTCTTCAGGAAC | TTCTCAAGGAAATTTTAGCAAATC |

**Table S2** Frequency distribution of clinicopathological features in breast cancer patients

| **Clinicopathological features** | | **Frequency (%)** |
| --- | --- | --- |
| Tumor size | <2 cm | 152 (33.1%) |
|  | ≥2 cm | 307 (66.9%) |
| Lymph node metastasis | No | 184 (40.1%) |
|  | Yes | 275 (59.9%) |
| Histological grade | SBR 1-2 | 244 (53.2%) |
|  | SBR 3 | 215 (46.8%) |
| Venous invasion | None–little | 292 (63.6%) |
|  | Moderate–severe | 167 (36.4%) |
| IHC results | ER (-) | 202 (44.0%) |
|  | ER (+) | 257 (56.0%) |
|  | PR (-) | 208 (45.3%) |
|  | PR (+) | 251 (54.7%) |
|  | Her-2 (-) | 330 (71.9%) |
|  | Her-2 (+) | 129 (28.1%) |
|  | Ki-67 ≥ 14% | 294 (64.1%) |
|  | Ki-67 < 14% | 165 (35.9%) |

IHC: Immunohistochemistry; ER: Estrogen receptor; PR: Progesterone receptor; Her-2: human epidermal growth factor receptor 2; SBR: Scarff, Bloom and Richardson tumor grade.
